# Novel model predicts diastolic cardiac dysfunction in type 2 diabetes

**DOI:** 10.1080/07853890.2023.2180154

**Published:** 2023-03-13

**Authors:** Mingyu Hao, Xiaohong Huang, Xueting Liu, Xiaokang Fang, Haiyan Li, Lingbo Lv, Liming Zhou, Tiecheng Guo, Dewen Yan

**Affiliations:** a Department of Endocrinology, Shenzhen Clinical Research Center for Metabolic Diseases, Shenzhen Second People’s Hospital, the First Affiliated Hospital of Shenzhen University, Health Science Center of Shenzhen University, Shenzhen, China; b Shenzhen Institutes of Advanced Technology, Chinese Academy of Sciences, Shenzhen, China; c Guangzhou Medical University, Guangzhou, China; d Chiwan Community Health Service Centre, Shenzhen, China

**Keywords:** Diabetic cardiomyopathy, type 2 diabetes, diastolic cardiac dysfunction, clinical predictive model

## Abstract

**Objective:**

Diabetes mellitus complicated with heart failure has high mortality and morbidity, but no reliable diagnoses and treatments are available. This study aimed to develop and verify a new model nomogram based on clinical parameters to predict diastolic cardiac dysfunction in patients with Type 2 diabetes mellitus (T2DM).

**Methods:**

3030 patients with T2DM underwent Doppler echocardiography at the First Affiliated Hospital of Shenzhen University between January 2014 and December 2021. The patients were divided into the training dataset (*n* = 1701) and the verification dataset (*n* = 1329). In this study, a predictive diastolic cardiac dysfunction nomogram is developed using multivariable logical regression analysis, which contains the candidates selected in a minor absolute shrinkage and selection operator regression model. Discrimination in the prediction model was assessed using the area under the receiver operating characteristic curve (AUC-ROC). The calibration curve was applied to evaluate the calibration of the alignment nomogram, and the clinical decision curve was used to determine the clinical practicability of the alignment map. The verification dataset was used to evaluate the prediction model’s performance.

**Results:**

A multivariable model that included age, body mass index (BMI), triglyceride (TG), creatine phosphokinase isoenzyme (CK-MB), serum sodium (Na), and urinary albumin/creatinine ratio (UACR) was presented as the nomogram. We obtained the model for estimating diastolic cardiac dysfunction in patients with T2DM. The AUC-ROC of the training dataset in our model was 0.8307, with 95% CI of 0.8109–0.8505. Similar to the results obtained with the training dataset, the AUC-ROC of the verification dataset in our model was 0.8083, with 95% CI of 0.7843–0.8324, thus demonstrating robust. The function of the predictive model was as follows: Diastolic Dysfunction = −4.41303 + 0.14100*Age(year)+0.10491*BMI (kg/m^2^) +0.12902*TG (mmol/L) +0.03970*CK-MB (ng/mL) −0.03988*Na(mmol/L) +0.65395 * (UACR > 30 mg/g) + 1.10837 * (UACR > 300 mg/g). The calibration plot diagram of predicted probabilities against observed DCM rates indicated excellent concordance. Decision curve analysis demonstrated that the novel nomogram was clinically useful.

**Conclusion:**

Diastolic cardiac dysfunction in patients with T2DM can be predicted by clinical parameters. Our prediction model may represent an effective tool for large-scale epidemiological study of diastolic cardiac dysfunction in T2DM patients and provide a reliable method for early screening of T2DM patients with cardiac complications.

**KEY MESSAGES**
This study used clinical parameters to predict diastolic cardiac dysfunction in patients with T2DM. This study established a nomogram for predicting diastolic cardiac dysfunction by multivariate logical regression analysis. Our predictive model can be used as an effective tool for large-scale epidemiological study of diastolic cardiac dysfunction in patients with T2DM and provides a reliable method for early screening of cardiac complications in patients with T2DM.

## Introduction

Type 2 diabetes mellitus (T2DM), complicated with heart failure (HF), has high mortality and morbidity. About 20% of T2DM patients have HF [1]. However, clinicians are currently very limited in treating T2DM with HF. Diabetes can cause myocardial ischemia and hypoxia changes in coronary arteries and other large vessels and directly cause myocardial metabolic changes in cardiomyocytes. As early as 1972, Rubber proposed that diabetic patients can cause cardiomyopathy without coronary artery ischemia [2]. The European Society of Cardiology defines diabetic cardiomyopathy (DCM) as cardiomyopathy with myocardial structural changes and ventricular systolic and diastolic dysfunction in patients with diabetes, excluding hypertensive heart disease, coronary heart disease, and cardiac valvular disease. DCM is the leading cardiovascular complication of diabetic patients. According to epidemiological reports, the incidence of diabetic cardiomyopathy is 10–21% [3], and the mortality rate of patients with diabetic cardiomyopathy is 31% [4]. The early stage of DCM is characterized by left ventricular hypertrophy, increased myocardial stiffness, increased ventricular filling pressure, and impaired diastolic function. In the late setting of DCM, cardiac fibrosis is aggravated, the diastolic function is further damaged, and secondary systolic dysfunction occurs [5,6]. DCM has no apparent symptoms in the early clinical stage and can only be diagnosed when the heart has a certain degree of dysfunction. If HF has occurred at the time of diagnosis, the function and structure of the myocardium cannot be reversed [4]. Before the development of symptomatic HF, as much as 50% of patients with T2DM develop asymptomatic left ventricular dysfunction [7]. In an early study, subclinical diastolic dysfunction in patients with diabetes was found to significantly increase the incidence of heart failure and mortality [8]. Therefore diastolic cardiac dysfunction in patients with T2DM is considered to be the clinical feature of DCM. So far, many factors have been proposed to contribute to DCM, such as glucose and lipid metabolism disorders, insulin resistance, oxidative stress, inflammatory response, mitochondrial disorders, endoplasmic reticulum stress, renin-angiotensin-aldosterone system, cardiac autonomic neuropathy, and cardiomyocyte apoptosis [9,10]. There is a clear clinical progression from DCM to HF. Myocardial cell dysfunction can cause myocardial fibrosis and remodeling, make the ventricle stiff, decrease compliance, further aggravate the diastolic cardiac dysfunction, and eventually develop into left ventricular ejection fraction preserved HF [11]. Currently, the clinical diagnosis of DCM is still challenging because researchers have not yet understood its pathogenesis and most patients with DCM have no symptoms before cardiac function deteriorates. Therefore, it is critical to study the clinical characteristics and risk factors of diastolic cardiac dysfunction in patients with diabetes to develop a new model to evaluate diastolic cardiac dysfunction in T2DM and provide a reliable basis for early diagnosis of DCM.

## Method

### Patients and study design

**Figure 1.**
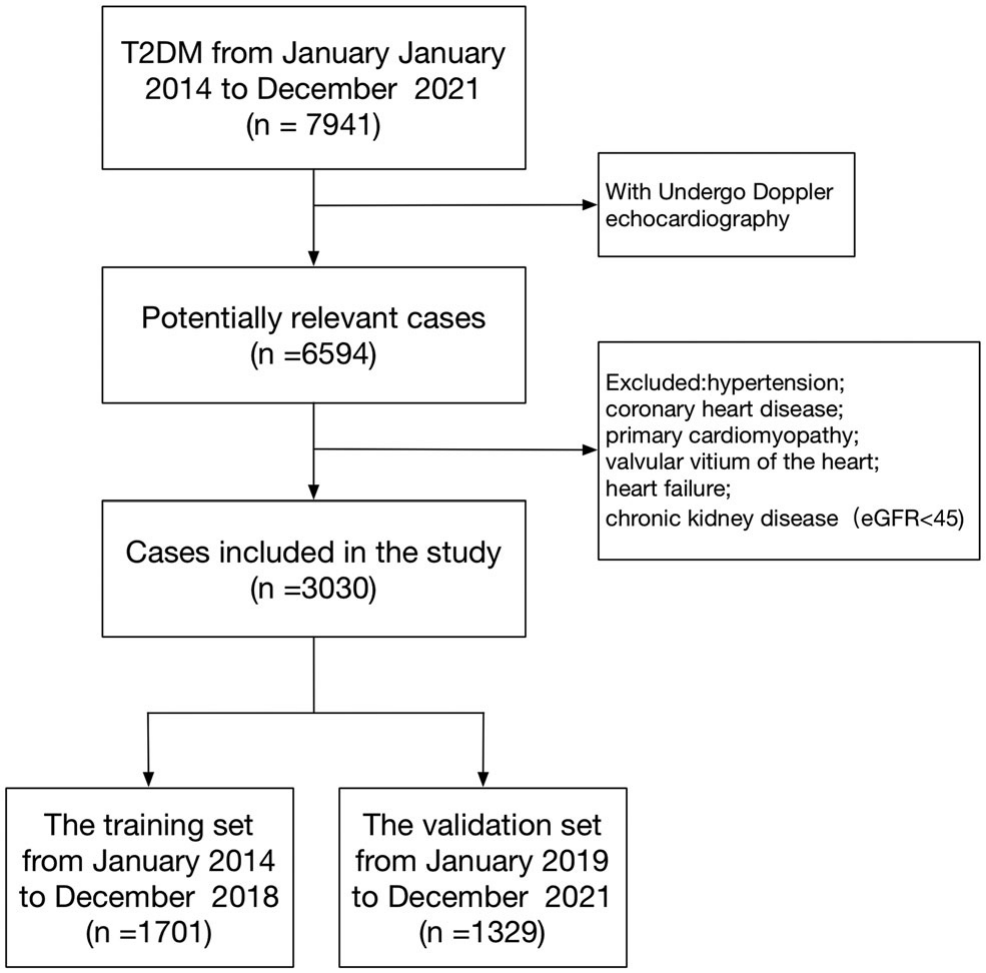
Flowchart of study participants.

This research was a retrospective analysis of patients with T2DM at the First Affiliated Hospital of Shenzhen University between January 2014 and December 2021. The data came from the electronic medical record system. The inclusion criteria were as follows: T2DM patients with DCM. T2DM was diagnosed according to the 2022 American Diabetes Association (ADA) criteria [12], that is, glycated hemoglobin A1c (HbA1c) ≥ 6.5%, and (or) fasting glucose ≥7.0, and (or) 2 h plasma glucose ≥ 11.1 mmol/L during oral glucose tolerance test. The diagnosis of DCM is based on Doppler echocardiography in T2DM patients with diastolic dysfunction. The exclusion criteria are as follows: the patients with hypertension, coronary heart disease, thyroid disease, chronic kidney disease, rheumatic heart disease, primary cardiomyopathy, valvular heart disease, and heart failure. The screening flow chart of the participants in this study is shown in Figure 1. The training dataset (January 2014 to December 2018) and the verification dataset (January 2019 to December 2021) were generated from the study population. The training dataset was used to establish the model, and the verification dataset was used to evaluate the preliminary performance of the model independently.

### Clinical and laboratory data

The clinical and laboratory data were colllected including age, gender, body mass index (BMI), course of diabetes (from diagnosis to hospitalization), smoking, drinking, diabetic complications, glycosylated haemoglobin (HbA1c), fasting blood glucose (FBG), postprandial blood glucose (PBG), fasting insulin (INS), postprandial insulin (INS-2h), fasting C peptide (CP), postprandial C peptide (CP-2h), triglyceride (TG), total cholesterol (TC), low-density lipoprotein cholesterol (LDL), high-density lipoprotein cholesterol (HDL), homocysteine (HCY), troponin I (cTnI), Myoglobin (Moy), creatine phosphokinase isoenzyme (CK-MB), urea nitrogen (BUN), carbon dioxide binding capacity (CO_2_CP), Cystatin C (CysC), retinol-binding protein (RBP), uric acid (UA),serum potassium (K), serum sodium (Na), serum magnesium (Mg), serum calcium (Ca), serum phosphorus (P), serum total protein levels (TP), total bilirubin (TBIL), fibronectin (FN), alanine aminotransferase (ALT), glutamyl transpeptidase (GGT), urinary albumin (UAL), Urine N-acetyl-β-D-glucosaminidase (UNAG), urinary albumin/creatinine ratio (UACR). The estimated GFR (eGFR) was calculated according to the CKD-EPI Epidemiology of Chronic Kidney Disease [13]. The blood glucose was detected by the enzyme electrode method using the Biosen5030 automatic blood glucose analyzer; The glycosylated hemoglobin was detected by high-pressure liquid chromatography with the Bole D10 glycosylated hemoglobin detector; Using Hitachi 7600-020 and Olympus 5421 automatic biochemical analyzer to detect blood lipids, serum creatinine, serum uric acid and urine albumin through routine biochemical colorimetry; Philips epiq-7c color ultrasound was used to detect cardiac color ultrasound. The laboratory and ultrasonic department provide data, and the instruments and staff are fixed. All results are reviewed during the inspection to ensure the availability and consistency of data.

### Doppler echocardiography

Doppler echocardiography was performed to determine left ventricular functional parameters by color ultrasound, and left ventricular ejection fraction (LVEF) was obtained by M-mode echocardiography and apical four-chamber view. The parameters of early diastolic peak velocity (E) and late diastolic peak velocity (A) were measured, and the ratio of E/A was calculated as the ultrasonic diagnostic criteria of diastolic cardiac dysfunction [14].

### Statistical analyses

Statistical analyses were performed using R statistical software (version 4.2.0) and IBM SPSS Statistics (version 22). Two-tailed *p*-value< 0.05 were considered statistically significant. The number of participants with missing data of BMI, TG, CK-MB, Na and UAER was 24 (0.8%), 93 (3.1%), 368 (12.1%), 22 (0.7%) and 623 (20.5%), respectively. Multiple imputations were used to handle the missing data of covariants. Missing data analysis procedures use missing-at-random (MAR) assumptions [15,16]. In this study, categorical and continuous variables were expressed as frequency (percentage, %) and mean (*SD*) or median (interquartile interval, IQR). This study uses the Student *t*-test or non-parametric Mann-Whitney *U* test for continuous variables. The *χ*^2^ Fisher exact test was used to evaluate the difference in baseline characteristics between the training and validation dataset.

The minor absolute shrinkage and selection operator (LASSO) method is suitable for reducing high-dimensional data [17,18] and was used to select the most useful predictive candidates from the training dataset. Candidates with non-zero coefficients are chosen to establish the LASSO model [19]. Multivariable logistic regression analysis to screen for independent clinical predictors related to DCM. Calculate each candidate’s OR, 95% CI, and *p*-value to predict possible diagnosis. In multivariate analysis, nomographs are generated based on these risk factors. The area under the receiver operating characteristic curve (AUC-ROC) is used to estimate the accuracy and discrimination of the nomographs and these scoring systems in the training and validation dataset. The nomogram provided a quantitative tool to predict the individual probability of diastolic dysfunction diagnosis.

The calibration curve is the consistency between the frequency of observed results and prediction probability. Research calibration is expressed by following the relationship between the frequency of the effect and the predicted probability. A sensible calibration measure is a likelihood ratio statistic testing the null hypothesis that intercept = 0 and slope = 1. The statistic has a *χ*^2^ distribution with 2 degrees of freedom (unreliability U-statistic) [20]. We also evaluated average (E-aver) and maximal errors (E-max) between predictions and observations obtained from a calibration curve. Plotted the calibration curve to assess the calibration of the nomogram, and the nonsignificant test statistics show that the model has been perfectly calibrated [21]. Decision curve analysis (DCA) was used to evaluate the clinical value of the predictive model. Decision curve analysis was conducted to determine the clinical usefulness of the nomogram by quantifying the net benefits at different threshold probabilities in the validation dataset [22].

### Ethical standards

The procedure followed in this study was in line with the standards established by the Ethics Committee of the First Affiliated Hospital of Shenzhen University. Approved by the ethics committee, and the ethics number is 20220209005. Since the previously collected clinical data could not contact the patient, he applied for an exemption from signing the informed consent form during the ethical review. The patient’s personal information has been desensitized and kept secret, and the research project does not involve personal privacy and commercial interests. The study results of this project may be published in medical journals. Still, we will keep the patient’s information confidential following the requirements of the law, and the patient’s personal information will not be disclosed unless relevant laws require it. Government administrative departments, hospital ethics committees, and appropriate personnel may consult patients’ data per the regulations.

## Results

### General information on patients

A total of 7941 patients with T2DM were initially enrolled in this study between January 2014 and December 2021. Of them, 6594 patients received Doppler echocardiography, and 3030 patients were eligible for this study. The median age of these eligible patients was 55 years (IQR 47–62). Among them, 1999 (65.97%) patients were male, and 1050 (34.7%) patients had a disease course for more than ten years. The median BMI was 24.2 (IQR 22.0–26.3) kg/m^2^, and the median HbA1c was 8.6%. (IQR 7.0 − 10.7%) There were 1925 (63.53%) patients with diastolic cardiac dysfunction. The patients were assigned to two study groups: the training dataset (January 2014 to December 2018) and the validation dataset (January 2019 to December 2021). The training and validation dataset had 1701 and 1329 T2DM patients, respectively. There were 1086 (63.84%) patients with diastolic cardiac dysfunction in the training dataset versus 839 (63.13%) patients in the validation dataset. The baseline characteristics of the two groups were similar, as shown in Table 1.

**Table 1.** The baseline characteristics of T2DM patients in the training and validation sets.

Clinical parameters	Training dataset (*n* = 1701)	Validation dataset (*n* = 1329)	Standardize diff.	*p*-Value
Age, years	55.00 (48.00–63.00)	53.00 (45.00–62.00)	0.18 (0.11, 0.25)	<0.001
Gender			0.04 (−0.03, 0.11)	0.272
Male	1108 (65.14%)	891 (67.04%)		
Female	593 (34.86%)	438 (32.96%)		
BMI, kg/m^2^	24.16 (22.10–26.20)	24.30 (22.00–26.40)	0.01 (−0.06, 0.08)	0.714
Smoking			0.01 (−0.06, 0.08)	0.848
No	1095 (64.37%)	860 (64.71%)		
Yes	606 (35.63%)	469 (35.29%)		
Drinking			0.02 (−0.06, 0.09)	0.680
No	1277 (75.07%)	989 (74.42%)		
Yes	424 (24.93%)	340 (25.58%)		
Diabetes duration			0.10 (0.03, 0.17)	0.119
<1 year	196 (11.52%)	193 (14.52%)		
1–5 years	412 (24.22%)	321 (24.15%)		
5–10 years	496 (29.16%)	362 (27.24%)		
10–20 years	486 (28.57%)	358 (26.94%)		
>20 years	111 (6.53%)	95 (7.15%)		
Diabetic complications			0.10 (0.03, 0.17)	0.008
No	789 (46.38%)	552 (41.53%)		
Yes	912 (53.62%)	777 (58.47%)		
HbA1c, %	8.60 (7.00–10.60)	8.60 (7.00–10.80)	0.02 (−0.05, 0.09)	0.874
FBG, mmol/L	7.00 (5.60–9.22)	6.42 (5.23–8.05)	0.26 (0.18, 0.33)	<0.001
PBG, mmol/L	15.50 (12.22–18.71)	15.76 (12.28–19.44)	0.06 (−0.01, 0.14)	0.087
INS, μIU/mL	8.80 (4.93–14.28)	8.07 (4.41–13.52)	0.08 (0.01, 0.15)	0.008
INS-2h, μIU/mL	23.08 (14.31–37.25)	25.06 (15.72–40.35)	0.07 (−0.01, 0.14)	0.009
CP, ng/ mL	1.34 (0.86–2.03)	1.83 (1.13–2.75)	0.25 (0.18, 0.32)	<0.001
CP-2h, ng/ mL	3.42 (2.18–5.57)	5.72 (3.29–8.88)	0.60 (0.52, 0.67)	<0.001
TG, mmol/L	1.45 (1.00–2.18)	1.54 (1.02–2.30)	0.12 (0.05, 0.19)	0.045
TC, mmol/L	4.40 (3.77–5.06)	4.39 (3.66–5.14)	0.01 (−0.06, 0.08)	0.695
LDL, mmol/L	2.70 (2.19–3.23)	2.77 (2.15–3.37)	0.08 (0.01, 0.15)	0.070
HDL, mmol/L	1.08 (0.93–1.27)	1.03 (0.88–1.21)	0.24 (0.17, 0.31)	<0.001
HCY, μmol/L	10.51 (8.60–12.70)	9.10 (7.50–11.30)	0.24 (0.16, 0.31)	<0.001
cTnI			0.69 (0.61, 0.76)	<0.001
<0.001, ng/ mL	720 (42.33%)	176 (13.24%)		
≥0.001, ≤0.01, ng/ mL	955 (56.14%)	1126 (84.73%)		
>0.01, ng/ mL	26 (1.53%)	27 (2.03%)		
Moy, ng/ mL	16.11 (11.70–24.10)	38.00 (23.00–55.00)	0.86 (0.79, 0.94)	<0.001
CK-MB, ng/ mL	1.48 (0.91–2.47)	2.00 (2.00–2.43)	0.11 (0.04, 0.18)	<0.001
BUN, mmol/L	4.90 (4.00–5.80)	5.30 (4.40–6.40)	0.31 (0.23, 0.38)	<0.001
CO_2_CP, mmol/L	26.60 (24.50–28.50)	26.00 (24.20–28.00)	0.12 (0.04, 0.19)	<0.001
CysC, mg/L	0.75 (0.63–0.90)	0.82 (0.71–0.94)	0.29 (0.22, 0.37)	<0.001
RBP, mg/L	43.60 (36.60–49.60)	39.60 (32.70–48.00)	0.20 (0.13, 0.28)	<0.001
UA, μmol/L	347.00 (287.50–411.60)	342.50 (286.30–408.50)	0.03 (−0.05, 0.10)	0.241
eGFR, mL/min	108.05 (97.87–117.00)	109.83 (99.16–120.76)	0.14 (0.07, 0.21)	<0.001
K, mmol/L	4.01 (3.83–4.20)	4.05 (3.84–4.26)	0.06 (−0.02, 0.13)	0.155
Na, mmol/L	139.60 (137.90–141.20)	139.60 (138.30–140.90)	0.07 (−0.01, 0.14)	0.673
Mg, mmol/L	0.89 (0.83–0.94)	0.86 (0.80–0.92)	0.30 (0.22, 0.37)	<0.001
Ca, mmol/L	2.24 (2.17–2.31)	2.22 (2.15–2.30)	0.17 (0.10, 0.24)	<0.001
P, mmol/L	1.22 (1.11–1.35)	1.25 (1.11–1.38)	0.08 (0.01, 0.15)	0.018
TP, g/L	66.80 (63.60–70.20)	66.30 (63.20–69.65)	0.10 (0.03, 0.17)	0.010
TBIL, μmol/L	10.50 (8.10–13.50)	10.30 (8.00–13.30)	0.04 (−0.03, 0.11)	0.141
FN, mg/L	207.00 (189.00–228.00)	234.00 (203.00–293.00)	0.73 (0.66, 0.81)	<0.001
ALT, U/L	17.20 (12.40–27.00)	17.00 (12.00–26.30)	0.01 (−0.06, 0.08)	0.449
GGT, U/L	25.00 (17.00–38.00)	24.00 (16.00–37.00)	0.01 (−0.06, 0.08)	0.014
UAL, mg/L	6.30 (3.10–13.90)	6.30 (3.40–14.00)	0.01 (−0.06, 0.08)	0.433
UNAG, U/L	7.00 (4.00–11.00)	8.00 (4.40–13.00)	0.15 (0.07, 0.22)	0.007
UACR			0.03 (−0.04, 0.10)	0.737
≤30, mg/g	1339 (78.72%)	1055 (79.38%)		
>30, ≤300, mg/g	308 (18.11%)	228 (17.16%)		
>300, mg/g	54 (3.17%)	46 (3.46%)		
Diastolic dysfunction			0.01 (−0.06, 0.09)	0.685
No	615 (36.16%)	490 (36.87%)		
Yes	1086 (63.84%)	839 (63.13%)		

### LASSO regression in the training dataset

**Figure 2.**
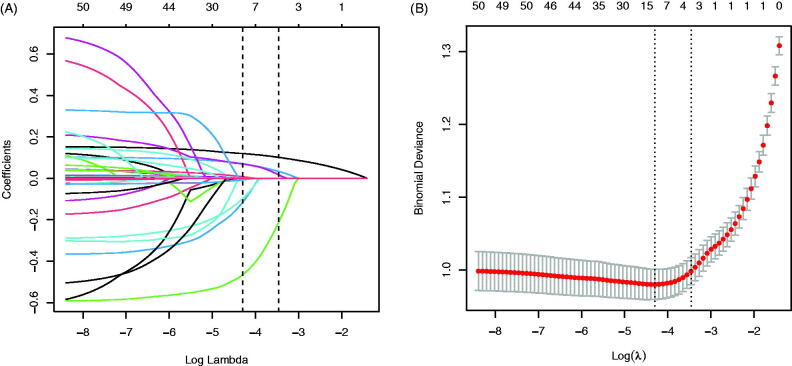
Demographic and clinical feature selection using the LASSO binary logistic regression model. (A) Optimal candidate (Lambda) selection in the LASSO model used 10-fold cross-validation *via* minimum criteria. The area under the receiver operating characteristic curve was plotted versus log (Lambda). Dotted vertical lines were drawn at the optimal values by using the minimum criteria and the 1 SE of the minimum standards; (B) LASSO coefficient profiles of the 41 candidates. A coefficient profile plot was produced against the log (Lambda) sequence. A vertical line was drawn at the value selected using 10-fold cross-validation, where optimal Lambda resulted in 9 candidates with non-zero coefficients (Lambda = 0.0136).

In the training dataset, 41 risk factors of the patient’s demographic, clinical, and laboratory indicators were included in the LASSO regression analysis (Figure 2). The variable with a non-zero coefficient in the LASSO regression model is considered related to DCM. They were selected for further research, including age, diabetes duration, BMI, TG, Myo, CK-MB, Na, FN, and UACR. The value of Lambda that the minimum mean cross-validated error is 0.0136.

### Development of an individualized prediction Model

**Figure 3.**
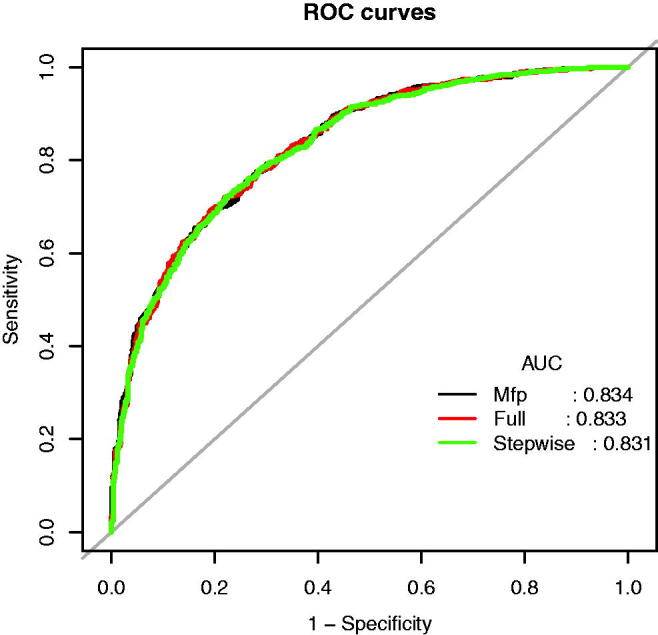
The ROC curves of the Model MFP (AUC-ROC is 0.834), model full (AUC-ROC is 0.833), and Model Stepwise (AUC-ROC is 0.831).

Using the predictors screened by Lasso regression as independent variables, we constructed three multivariate logistic regression models, the Multiple Fractional Polynomial (MFP) model, the Full model, and the Stepwise model. These three models’ areas under the receiver operating characteristic curve (AUC-ROC) were relatively close. The AUC-ROC, respectively, were 0.8336, 0.8327, and 0.8307 for these three models (Figure 3). The analysis results of the three models were compared in Table 2. Given that the Stepwise model incorporated fewer risk factors and the Akaike Information Criterion (AIC) value is minimum and could predict diastolic cardiac dysfunction in patients with Type 2 diabetes mellitus relatively well, we choose the Stepwise model as the final risk prediction model for diastolic cardiac dysfunction in patients with Type 2 diabetes mellitus. The results of multivariable logistic regression analyses are shown in Table 3.

**Table 2.** Prediction performance the Model MFP, Model Full, and Model Stepwise for the risk of impaired diastolic function in T2DM patients.

Test	MFP	Full	Stepwise
1	1086	1086	1086
0	615	615	615
ROC area (AUC)	0.8336	0.8327	0.8307
95% CI	0.8141–0.8531	0.8130–0.8523	0.8109–0.8505
Best threshold	0.6765	0.6510	0.6506
Specificity	0.8049	0.7805	0.7789
Sensitivity	0.6924	0.7192	0.7192
Accuracy	0.7331	0.7413	0.7407
Positive-LR	3.5488	3.2761	3.2521
Negative-LR	0.3821	0.3598	0.3606
Diagnose-OR	9.2874	9.1046	9.0188
N-for-diagnose	2.0107	2.0014	2.0080
Postive-pv	0.8624	0.8526	0.8517
Negative-pv	0.5971	0.6115	0.6110
a	752	781	781
b	120	135	136
c	334	305	305
d	495	480	479

**Table 3.** Multivariable analyses of impaired diastolic function in T2DM patients in the training set.

	Estimate	Std error	OR	95% CI	*p*-Value
(Intercept)	−4.4130	3.3615	0.0121	0.0000–8.8075	0.1893
Age, years	0.1410	0.0078	1.1514	1.1340–1.1691	0.0000
BMI, kg/m^2^	0.1049	0.0196	1.1106	1.0687–1.1542	0.0000
TG, mmol/L	0.1290	0.0441	1.1377	1.0435–1.2405	0.0034
CK-MB, ng/ mL	0.0397	0.0250	1.0405	0.9907–1.0928	0.1125
Na, mmol/L	−0.0399	0.0237	0.9609	0.9174–1.0065	0.0919
UACR > 30, mg/g	0.6540	0.1743	1.9231	1.3665–2.7065	0.0002
UACR > 300, mg/g	1.1084	0.4063	3.0294	1.3663–6.7172	0.0064

**Figure 4.**
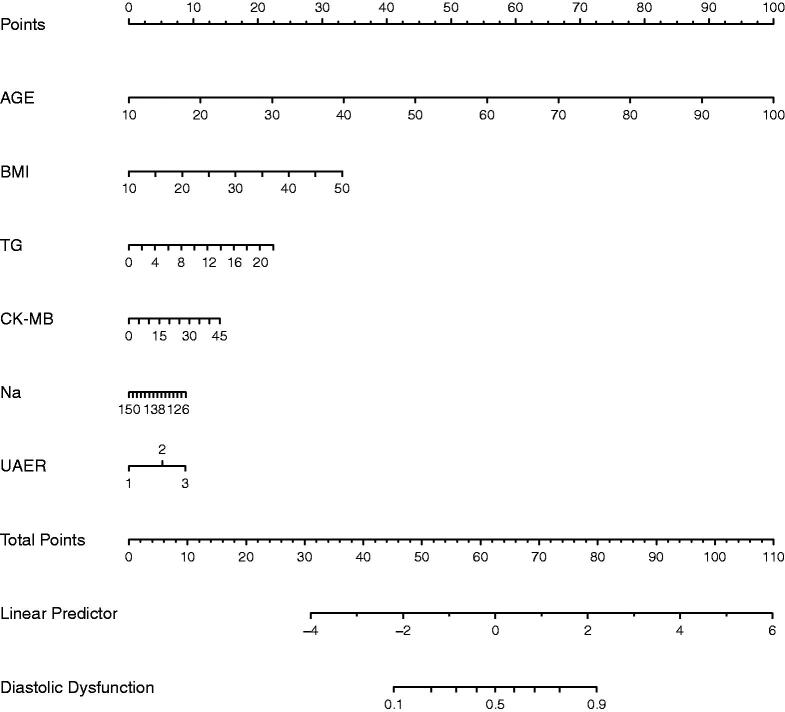
Nomogram predicting DN. The nomogram was developed in the training dataset with AGE, BMI, TG, CK-MB, Na, and UAER. Points of each variable were acquired by drawing a straight line upward from the corresponding value to the ‘Points’ line. Then sum the points received from each variable and locate the number on the ‘Total Points’ axis. To conclude the patient’s sort probability of having diastolic dysfunction, draw a straight line down to the corresponding ‘Probability of Diastolic Dysfunction’ axis. Units: AGE, years; BMI, kg/m^2^; TG, mmol/L; CK-MB, ng/mL; Na, mmol/L; UAER, mg/g.

**Figure 5.**
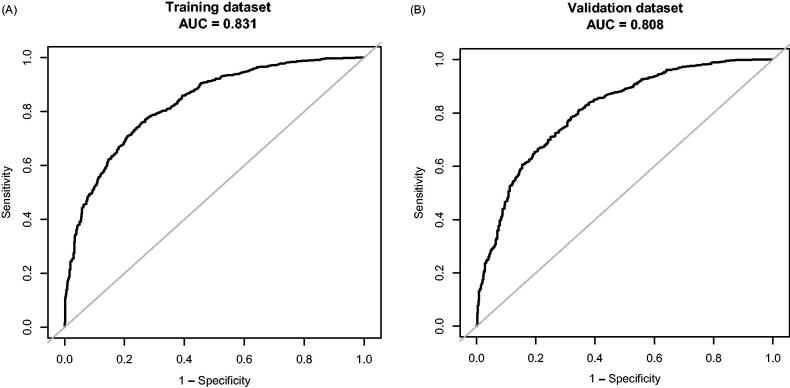
The ROC curves of the Novel model in the training dataset (A) and validation dataset (B).

Multivariable logistic regression analysis demonstrated that six variables (Age, BMI, TG, CK-MB, Na, and UACR) are independently associated with DCM. This research developed a model incorporating the six potential predictors and presented it as a nomogram (Figure 4). The nomogram was assigned a specific score, and the total score was used to obtain the probability of predicting DCM. The ratios of the calculated beta were used to evaluate the proportional predictive effects of these variables. The projections from total points on the scales below indicated the estimated probability of DCM. Therefore, the best prediction model we proposed was as follows: Diastolic Dysfunction= −4.41303 + 0.14100*Age (year)+0.10491*BMI (kg/m^2^) +0.12902*TG (mmol/L) +0.03970*CK-MB (ng/mL)−0.03988*Na(mmol/L) +0.65395 * (UACR > 30 mg/g) + 1.10837 * (UACR > 300 mg/g). Training dataset, the area under the receiver operating characteristic curve (AUC-ROC) in our model is 0.831 (95% CI, 0.8109 − 0.8505) (Figure 5(A)). Bring the validation dataset into the regression equation. The area under the receiver operating characteristic curve (AUC-ROC) is 0.808 (95% CI, 0.7843 − 0.8324) (Figure 5(B)). Other analysis results are shown in Table 4. To verify the important role of these risk factors in diastolic cardiac dysfunction, we provide results for the linear regression of predicting specific indices of cardiac dysfunction, including right ventricular outflow tract(RVOT), left atrium(LA), left ventricular outflow tract(LOVT), right ventricle(RV), interventricular septum(IVS), left ventricular diameter(LVD), left ventricular ejection fraction(EF), E/A ratio, pulmonary vein(PV), pulmonary artery velocity(VPA). In supplementary Tables 1–10.

**Table 4.** Prediction performance of the nomogram for the risk of impaired diastolic function in T2DM patients in the training and validation sets.

Test	Training dataset	Validation dataset
1	1086	839
0	615	490
ROC area(AUC)	0.8307	0.8083
95% CI	0.8109–0.8505	0.7843–0.8324
Best threshold	0.6506	0.5532
Specificity	0.7789	0.6551
Sensitivity	0.7192	0.8117
Accuracy	0.7407	0.7540
Positive-LR	3.2521	2.3534
Negative-LR	0.3606	0.2875
Diagnose-OR	9.0188	8.1867
N-for-diagnose	2.0080	2.1423
Postive-pv	0.8517	0.8012
Negative-pv	0.6110	0.6701
a	781	681
b	136	169
c	305	158
d	479	321

### Apparent performance of the nomogram

**Figure 6.**
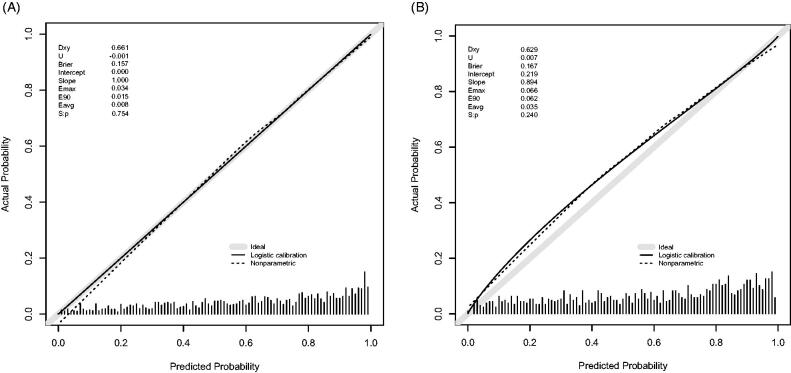
Calibration curve of the Novel model in the training dataset (A) and validation dataset (B). The *x*-axis represents the predicted probability of Diastolic Dysfunction. The *y*-axis represents the actual diagnosed Diastolic Dysfunction. The diagonal dotted line represents a perfect prediction by an ideal model. The solid line represents the performance of the nomogram, of which a closer fit to the diagonal dotted line means a better prognosis.

The calibration curve of the nomogram for the probability of diastolic cardiac dysfunction demonstrated good agreement between prediction and observation in the training dataset (Figure 6). The Hosmer-Lemeshow test indicated that the model was nonsignificant (*p* = 0.627, *p* > 0.05). These results show that the model fits the observed data perfectly. The average difference (E-aver) in predicted and calibrated probabilities in the training dataset is 0.008. The maximal difference (E-max) is 0.034. The *p-*value of the *U* index was 0.754, and the *p*-value obtained was as expected. The average difference (E-aver) in predicted and calibrated probabilities in the validation dataset is 0.035. The maximal difference (E-max) is 0.066. The *p*-value of the *U* index was 0.240, and the *p*-value is expected.

### Clinical use

**Figure 7.**
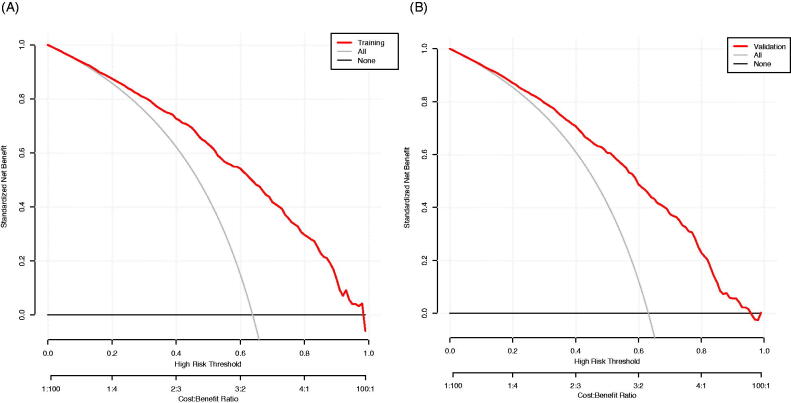
The decision curve analysis of the Novel model in the training dataset (A) and validation dataset (B). The black line represents the net benefit when none of the participants is considered to have Diastolic Dysfunction. In contrast, the light grey line represents the net benefit when all participants are deemed to have Diastolic Dysfunction. The area between the ‘no treatment line’ (black line) and ‘all treatment line’ (light grey line) in the model curve indicates the clinical utility of the model. The farther the model curve is from the black and light grey lines, the better the clinical use of the nomogram.

The result of the decision curve analysis for the nomogram is presented in Figure 7. In the training dataset, the decision curve showed that if the threshold probability of a patient was >1%, the net benefit was more than 90%. The above results show a broad spectrum of alternative threshold probability in the model, suggesting that the model was a good assessment tool. Therefore, we can use the nomogram to predict the diagnosis of diastolic cardiac dysfunction.

## Discussion

This study explored the clinical characteristics and risk factors of diastolic cardiac dysfunction in patients with diabetes and constructed and validated a new model to evaluate diastolic cardiac dysfunction in T2DM, which provided a reliable tool for early diagnosis of DCM. The diagnostic model included age, BMI, TG, CK-MB, Na, and UACR. From our research, the calibration chart showed good consistency between the actual and predictive diagnoses. Similarly, the validation dataset al.so showed satisfactory robustness. We did an ASCVD risk score on the data and compared it with our prediction model. The result shows that our model prediction is better than the ASCVD risk score. The comparison results of the two models are shown in Supplementary Table 11 and Supplementary Figure 1.

Left ventricular diastolic dysfunction indicates decreased diastolic compliance, which is related to cardiac tissue remodeling and is considered one of the early manifestations of DCM. Cardiac tissue Doppler showed that 60% of T2DM patients without hypertension and coronary heart disease had decreased left ventricular diastolic function [23], indicating that diastolic dysfunction was widespread in diabetic patients. In this study, ∼63% of T2DM patients showed left ventricular diastolic dysfunction, consistent with previous research results.

Many studies have shown that age is an independent risk factor for left ventricular diastolic dysfunction [24,25]. Age is the most important factor affecting diastolic cardiac dysfunction in patients with T2DM. In this study, the patients were divided into seven age groups, < 30 years old, 30–40 years old, 40–50 years old, 50–60 years old, 60–70 years old, 70–80 years old, and > 80 years old. The incidence of diastolic cardiac dysfunction in each age group was 7%, 16%, 42%, 69%, 88%, 92%, and 99%, respectively (*p* < 0.01). These results clearly show that the incidence of left ventricular diastolic dysfunction increases with age, which is consistent with the results of previous studies. Logistic regression results showed that the risk of diastolic cardiac dysfunction increased by one year with age (OR = 1.1514, 95% CI 1.1340 − 1.1691). Age is the most important risk factor in the pathogenesis of DCM, which not only directly affects the diastolic cardiac function but also indirectly leads to the decrease of diastolic cardiac function by affecting blood glucose, blood lipids, and Vascular endothelium [26,27].

In previous epidemiological investigations, 41% of diabetic patients were overweight and 24.3% obese in China [28]. In this study, 1214 (40%) were overweight (BMI ≥ 24), and 396 (13%) were obese (BMI ≥ 28), which was consistent with the data of the epidemic survey. Weight gain is an independent risk factor for T2DM. Weight gain can aggravate insulin resistance and increase the difficulty of blood glucose control [29]. Obese patients are often associated with insulin resistance and obesity, resulting in glucose, lipid metabolism disorders, and oxidative stress caused by vascular endothelial cell damage, which impacts the cardiovascular system [30]. This study analyzed the predictor of left ventricular diastolic dysfunction in patients with T2DM. BMI was independently related to left ventricular diastolic dysfunction (OR = 1.1106, 95% CI 1.0687 − 1.1542), consistent with previous studies in Asia [31], Europe [32], and America [33]. Our data further emphasize the importance of diet control and exercise health education in preventing diastolic cardiac dysfunction in T2DM patients.

The myocardium of patients with diabetes is powered by free fatty acids [34]. The overuse of fatty acids in the myocardium will lead to the accumulation of fatty acids in the myocardium and lipotoxicity. Free fatty acids are the intermediate products of triglyceride metabolism in the body. In this study, TG was independently associated with diastolic cardiac dysfunction (OR = 1.1377, 95% CI 1.0435 − 1.2405). Previous studies have shown that hypertriglyceridemia affects glucose regulation and insulin sensitivity [35], and both high glucose levels and insulin resistance play an essential role in the pathogenesis of DCM [36,37]. Therefore, as a risk factor of DCM, TG affects the deterioration of the disease, to which clinicians should pay more attention. Of note, TG often increases before the onset of T2DM. Therefore, monitoring the TG level may help predict the occurrence of diabetes and its complications.

DCM is the manifestation of diabetic microangiopathy in the myocardium, so diabetic nephropathy plays a hint role in the clinical diagnosis of DCM. UACR is a sensitive indicator of diabetic renal damage and is closely related to vascular endothelial dysfunction [38,39]. In clinic practice, UACR is often used to evaluate diabetic nephropathy. In this study, UACR > 30 mg/g (OR = 1.9231, 95% CI 1.3665 − 2.7065), UACR > 300 mg/g (OR = 3.0294, 95% CI 1.3663 − 6.7172). Our results suggest that the urinary protein/creatinine ratio can be used to predict left ventricular diastolic dysfunction in patients with T2DM. Much attention should be paid to the risk of left ventricular diastolic dysfunction when UACR exceeds 30 mg/g.

CK-MB is mainly distributed in myocardial tissue and is a marker for evaluating myocardial injury [40]. Recent studies have shown that the level of CK-MB is positively correlated with the decrease of left ventricular diastolic function, and the content of CK-MB in the blood is closely related to the degree of myocardial injury [41]. This study used CK-MB as a risk factor to affect diastolic cardiac dysfunction in T2DM patients. The common electrolyte disorder in patients with heart failure is hyponatremia [42], with an incidence of 5–30% [43]. Previous Studies have shown that the mortality and readmission rates of heart failure patients with hyponatremia are significantly higher than those without hyponatremia. Here our data show that increased or decreased? Serum sodium reduces the risk of diastolic cardiac dysfunction. Therefore, decreasing serum sodium in diabetic patients is essential for DCM heart failure.

Theoretically, metabolic factors such as blood sugar are the crucial factors of DCM, and previous studies have found that when HbA1c < 6%, the incidence of heart failure in T2DM patients is 0.23%, and when HbA1c > 10%, the incidence of heart failure is 1.19% [44]. Poor blood glucose control increases the risk of heart failure and affects the occurrence of DCM. Our study’s median HbA1c was 8.6% (IQR 7.0 − 10.7%). Although glycosylated hemoglobin, blood glucose, and other risk factors are not included in this study, the age included in this study impacts blood glucose, and poor blood glucose control will also affect the urinary albumin/creatinine ratio, triglyceride, and blood glucose affect each other. The blood glucose control of diabetic patients hospitalized in the endocrinology department is generally poor. The difference between groups is not apparent. The blood glucose data used in this study can only reflect blood sugar for a while, not enough to evaluate the overall blood glucose level. They can not accurately reflect the fluctuation of blood sugar. DCM is a long-term process that is constantly affected by blood sugar. Short-term blood sugar during hospitalization is not enough to remember the severity of the disease.

This study has the following advantages: firstly, our overall sample size is relatively large; secondly, our prediction model contains six clinical parameters that are relatively easy to obtain and can be used in large-scale clinical practice; thirdly, the clinical information of our covariates is complete; fourthly, we use multiple interpolation methods to deal with the missing data, reducing the waste of data resources and improving the research’s effectiveness and accuracy. Indeed, this study also has shortcomings: as a cross-sectional study, the collected clinical data are limited, and some clinical data can not be collected, such as waist-to-hip ratio, lifestyle, treatment, and so on. However, our predictive model has an excellent performance in internal and external validation, which shows that the line chart based on the existing six risk factors is highly generalized. The data of this study comes from one single hospital. In future studies, we will include more data from clinical research centers and conduct multicenter studies to improve the study’s accuracy.

## Conclusion

The predictive models in this study include six readily available clinical parameters, age, BMI, TG, CK-MB, Na, and UACR, and show high accuracy in the verification dataset. Our prediction model provides an effective tool for the clinical evaluation of diastolic cardiac dysfunction in patients with type 2 diabetes, which may help clinicians with the early diagnosis of DCM and the prevention of the severe consequences of disease deterioration.

## Supplementary Material

Supplemental Material

## Data Availability

Data is available on request from the authors. The data supporting this study’s findings are available from the corresponding author D Yan upon reasonable request.
