# Supplementary material for: Novel model predicts diastolic cardiac dysfunction in type 2 diabetes
Source: Ann Med. 2023 Mar 13;55(1):766–77. doi: 10.1080/07853890.2023.2180154 (PMC10798288; doi:10.1080/07853890.2023.2180154)
Supplement: Supplemental Material [file IANN_A_2180154_SM8122.docx]

supplementary table-1RVOT（right ventricular outflow tract）

|  | Coeff. | Se. | 95%CI.low | 95%CI.upp | P.value |
| --- | --- | --- | --- | --- | --- |
| Intercept | 20.9332 | 3.8154 | 13.4550 | 28.4113 | <0.0001 |
| AGE | 0.0144 | 0.0058 | 0.0029 | 0.0258 | 0.0138 |
| BMI | 0.2839 | 0.0204 | 0.2438 | 0.3239 | <0.0001 |
| TG | -0.0374 | 0.0387 | -0.1132 | 0.0383 | 0.3330 |
| CKMB | 0.0219 | 0.0285 | -0.0340 | 0.0778 | 0.4428 |
| Na | -0.0077 | 0.0270 | -0.0607 | 0.0453 | 0.7768 |
| UACR > 30 mg/g | -0.0598 | 0.1801 | -0.4127 | 0.2932 | 0.7400 |
| UACR > 300 mg/g | 0.8919 | 0.3824 | 0.1424 | 1.6414 | 0.0197 |

supplementary table-2 LA（left atrium）

|  | Coeff. | Se. | 95%CI.low | 95%CI.upp | P.value |
| --- | --- | --- | --- | --- | --- |
| Intercept | 17.2373 | 3.4824 | 10.4117 | 24.0628 | <0.0001 |
| AGE | 0.0348 | 0.0053 | 0.0243 | 0.0452 | <0.0001 |
| BMI | 0.3827 | 0.0186 | 0.3461 | 0.4192 | <0.0001 |
| TG | 0.0154 | 0.0353 | -0.0538 | 0.0846 | 0.6624 |
| CKMB | 0.0359 | 0.0260 | -0.0152 | 0.0870 | 0.1683 |
| Na | 0.0163 | 0.0247 | -0.0321 | 0.0646 | 0.5101 |
| UACR > 30 mg/g | 0.3500 | 0.1644 | 0.0279 | 0.6722 | 0.0333 |
| UACR > 300 mg/g | 1.3656 | 0.3490 | 0.6815 | 2.0497 | <0.0001 |

supplementary table-3 LVOT（left ventricular outflow tract）

|  | Coeff. | Se. | 95%CI.low | 95%CI.upp | P.value |
| --- | --- | --- | --- | --- | --- |
| Intercept | 15.7449 | 4.3331 | 7.2519 | 24.2378 | 0.0003 |
| AGE | -0.0322 | 0.0066 | -0.0452 | -0.0192 | <0.0001 |
| BMI | 0.1074 | 0.0232 | 0.0619 | 0.1529 | <0.0001 |
| TG | -0.0857 | 0.0439 | -0.1718 | 0.0003 | 0.0510 |
| CKMB | -0.0234 | 0.0324 | -0.0870 | 0.0401 | 0.4697 |
| Na | 0.0606 | 0.0307 | 0.0005 | 0.1208 | 0.0484 |
| UACR > 30 mg/g | -0.0703 | 0.2045 | -0.4712 | 0.3305 | 0.7310 |
| UACR > 300 mg/g | -0.6738 | 0.4343 | -1.5250 | 0.1774 | 0.1209 |

supplementary table-4 RV（right ventricle）

|  | Coeff. | Se. | 95%CI.low | 95%CI.upp | P.value |
| --- | --- | --- | --- | --- | --- |
| Intercept | 16.0951 | 3.0828 | 10.0529 | 22.1373 | <0.0001 |
| AGE | -0.0053 | 0.0047 | -0.0145 | 0.0040 | 0.2643 |
| BMI | 0.1407 | 0.0165 | 0.1083 | 0.1730 | <0.0001 |
| TG | -0.0152 | 0.0312 | -0.0764 | 0.0461 | 0.6277 |
| CKMB | 0.0542 | 0.0231 | 0.0090 | 0.0994 | 0.0189 |
| Na | 0.0136 | 0.0218 | -0.0293 | 0.0564 | 0.5350 |
| UACR > 30 mg/g | 0.0108 | 0.1455 | -0.2744 | 0.2960 | 0.9408 |
| UACR > 300 mg/g | 0.1846 | 0.3090 | -0.4210 | 0.7902 | 0.5502 |

supplementary table-5 IVS（interventricular septum）

|  | Coeff. | Se. | 95%CI.low | 95%CI.upp | P.value |
| --- | --- | --- | --- | --- | --- |
| Intercept | 5.2259 | 1.2314 | 2.8124 | 7.6394 | <0.0001 |
| AGE | 0.0158 | 0.0019 | 0.0121 | 0.0195 | <0.0001 |
| BMI | 0.0902 | 0.0066 | 0.0773 | 0.1031 | <0.0001 |
| TG | 0.0179 | 0.0125 | -0.0066 | 0.0423 | 0.1525 |
| CKMB | 0.0162 | 0.0092 | -0.0019 | 0.0342 | 0.0794 |
| Na | 0.0102 | 0.0087 | -0.0069 | 0.0273 | 0.2403 |
| UACR > 30 mg/g | 0.1374 | 0.0581 | 0.0235 | 0.2513 | 0.0181 |
| UACR > 300 mg/g | 0.5354 | 0.1234 | 0.2935 | 0.7773 | <0.0001 |

supplementary table-6 LVD (Left ventricular diameter)

|  | Coeff. | Se. | 95%CI.low | 95%CI.upp | P.value |
| --- | --- | --- | --- | --- | --- |
| Intercept | 32.5160 | 4.2495 | 24.1869 | 40.8450 | <0.0001 |
| AGE | -0.0086 | 0.0065 | -0.0214 | 0.0041 | 0.1853 |
| BMI | 0.2481 | 0.0228 | 0.2035 | 0.2927 | <0.0001 |
| TG | -0.0427 | 0.0431 | -0.1271 | 0.0417 | 0.3212 |
| CKMB | 0.0430 | 0.0318 | -0.0193 | 0.1053 | 0.1759 |
| Na | 0.0531 | 0.0301 | -0.0059 | 0.1121 | 0.0780 |
| UACR > 30 mg/g | -0.5152 | 0.2006 | -0.9083 | -0.1221 | 0.0102 |
| UACR > 300 mg/g | 0.4397 | 0.4259 | -0.3951 | 1.2745 | 0.3020 |

supplementary table-7 EF

|  | Coeff. | Se. | 95%CI.low | 95%CI.upp | P.value |
| --- | --- | --- | --- | --- | --- |
| Intercept | 60.8529 | 5.1624 | 50.7346 | 70.9711 | <0.0001 |
| AGE | 0.0046 | 0.0079 | -0.0109 | 0.0200 | 0.5646 |
| BMI | -0.0538 | 0.0276 | -0.1079 | 0.0004 | 0.0518 |
| TG | -0.0229 | 0.0523 | -0.1254 | 0.0796 | 0.6617 |
| CKMB | 0.0403 | 0.0386 | -0.0354 | 0.1160 | 0.2970 |
| Na | 0.0461 | 0.0366 | -0.0256 | 0.1178 | 0.2073 |
| UACR > 30 mg/g | 0.0813 | 0.2436 | -0.3963 | 0.5588 | 0.7387 |
| UACR > 300 mg/g | -0.3269 | 0.5174 | -1.3410 | 0.6872 | 0.5275 |

supplementary table-8 E/A ratio

|  | Coeff. | Se. | 95%CI.low | 95%CI.upp | P.value |
| --- | --- | --- | --- | --- | --- |
| Intercept | 2.3764 | 0.3308 | 1.7280 | 3.0248 | <0.0001 |
| AGE | -0.0145 | 0.0005 | -0.0155 | -0.0135 | <0.0001 |
| BMI | -0.0130 | 0.0018 | -0.0165 | -0.0096 | <0.0001 |
| TG | -0.0069 | 0.0034 | -0.0135 | -0.0004 | 0.0389 |
| CKMB | -0.0013 | 0.0025 | -0.0062 | 0.0035 | 0.5891 |
| Na | -0.0016 | 0.0023 | -0.0062 | 0.0030 | 0.4911 |
| UACR > 30 mg/g | -0.0420 | 0.0156 | -0.0726 | -0.0114 | 0.0071 |
| UACR > 300 mg/g | -0.1248 | 0.0332 | -0.1897 | -0.0598 | 0.0002 |

supplementary table-9 PV（pulmonary vein）

|  | Coeff. | Se. | 95%CI.low | 95%CI.upp | P.value |
| --- | --- | --- | --- | --- | --- |
| Intercept | 16.4064 | 4.8189 | 6.9614 | 25.8515 | 0.0007 |
| AGE | 0.0054 | 0.0074 | -0.0090 | 0.0199 | 0.4618 |
| BMI | 0.1341 | 0.0258 | 0.0836 | 0.1847 | <0.0001 |
| TG | -0.0042 | 0.0488 | -0.0999 | 0.0915 | 0.9308 |
| CKMB | -0.0219 | 0.0360 | -0.0926 | 0.0487 | 0.5435 |
| Na | 0.0135 | 0.0341 | -0.0534 | 0.0804 | 0.6931 |
| UACR > 30 mg/g | 0.0508 | 0.2274 | -0.3949 | 0.4966 | 0.8231 |
| UACR > 300 mg/g | -0.0436 | 0.4830 | -0.9902 | 0.9031 | 0.9281 |

supplementary table-10 VPA(Pulmonary artery velocity)

|  | Coeff. | Se. | 95%CI.low | 95%CI.upp | P.value |
| --- | --- | --- | --- | --- | --- |
| Intercept | 68.5720 | 18.8135 | 31.6977 | 105.4464 | 0.0003 |
| AGE | -0.0375 | 0.0288 | -0.0940 | 0.0190 | 0.1931 |
| BMI | 0.4354 | 0.1007 | 0.2380 | 0.6328 | <0.0001 |
| TG | -0.3667 | 0.1906 | -0.7403 | 0.0070 | 0.0545 |
| CKMB | 0.2870 | 0.1407 | 0.0112 | 0.5629 | 0.0415 |
| Na | 0.0743 | 0.1333 | -0.1870 | 0.3356 | 0.5773 |
| UACR > 30 mg/g | 1.4575 | 0.8879 | -0.2828 | 3.1979 | 0.1008 |
| UACR > 300 mg/g | 6.8169 | 1.8856 | 3.1211 | 10.5126 | 0.0003 |

supplementary table-11 Compare 2 predictive models (ROC Curve)

| Test | Model1 | Model2 | P(compare) |
| --- | --- | --- | --- |
| 1 | 1925 | 1925 |  |
| 0 | 1105 | 1105 |  |
| ROC area(AUC) | 0.8174 | 0.6083 | 0.0000 |
| 95%CI low | 0.8019 | 0.5953 |  |
| 95%CI upp | 0.8328 | 0.6214 |  |
| Best threshold | 0.6886 | -0.1179 |  |
| Specificity | 0.7801 | 0.2407 |  |
| Sensitivity | 0.6971 | 0.9756 |  |
| Accuracy | 0.7274 | 0.7076 |  |
| Positive-LR | 3.1701 | 1.2849 |  |
| Negative-LR | 0.3882 | 0.1014 |  |
| Diagnose-OR | 8.1655 | 12.6683 |  |
| N-for-diagnose | 2.0954 | 4.6230 |  |
| Positive-pv | 0.8467 | 0.6912 |  |
| Negative-pv | 0.5965 | 0.8498 |  |
| a | 1342 | 1878 |  |
| b | 243 | 839 |  |
| c | 583 | 47 |  |
| d | 862 | 266 |  |

Supplementary Figure-1:model 1 is the AUC-ROC for our prediction model, and model 2 is the AUC-ROC for the ASCVD risk score.
